# Circular RNA FUNDC1 for Prediction of Acute Phase Outcome and Long-Term Survival of Acute Ischemic Stroke

**DOI:** 10.3389/fneur.2022.846198

**Published:** 2022-06-03

**Authors:** Juan Zu, Lei Zuo, Lin Zhang, Zan Wang, Yachen Shi, Lihua Gu, Zhijun Zhang

**Affiliations:** ^1^Department of Neurology, Key Laboratory of Developmental Genes and Human Disease, Affiliated Zhongda Hospital, School of Medicine, Institution of Neuropsychiatry, Southeast University, Nanjing, China; ^2^Department of Mental Health and Public Health, Faculty of Life and Health Sciences, Shenzhen Institute of Advanced Technology, Chinese Academy of Sciences, Shenzhen, China

**Keywords:** acute ischemic stroke, biomarker, circular RNA, early neurological deterioration, outcome

## Abstract

Circular RNAs (CircRNAs) have shown promising potential in the diagnosis and the prediction of outcomes of stroke. This study aimed to explore the potential value of circRNAs for identifying acute neurological deterioration and estimating long-term survival for acute ischemic stroke (AIS). One hundred healthy controls and 200 patients with AIS within 72 h were recruited, 140 of whom were admitted within 24 h after onset. CircRNA levels in peripheral blood were measured by quantitative polymerase chain reaction (qPCR). Compared to the controls, the levels of three circRNAs were significantly increased in three subgroups of patients, including large artery atherosclerosis (LAA) stroke, small artery occlusion (SAO) stroke, and cardioembolism (CE) stroke (all *P* < 0.001). Among, LAA stroke patients had higher levels of circular RNA FUNDC1 (circFUNDC1) compared to SAO stroke patients (*P* = 0.015). CircFUNDC1 levels were positively correlated with National Institutes of Health Stroke Scale (NIHSS) scores on the 7th day only in LAA patients (*P* = 0.048, *r* = 0.226). It should be noted that the levels of circFUNDC1 in patients with early neurological deterioration (END), admitted within 24 h after onset, were significantly higher than those without END (*P* = 0.013). In addition, circFUNDC1 levels positively correlated with baseline NIHSS scores (*P* = 0.016, *r* = 0.203) or the 7th day NIHSS scores (*P* = 0.001, *r* = 0.289) in patients within 24 h after onset. Importantly, after 18 months of follow-up, a significant difference was observed on survival Kaplan-Meier curves (*P* = 0.042) between AIS patients with low (below cut-off) or high circFUNDC1 levels (above cut-off). Circulating circFUNDC1 could be a potential biomarker for predicting acute-phase outcome and long-term survival in AIS.

## Introduction

Stroke is a common cause of mortality and disability worldwide. Although age-standardised stroke mortality has declined, the absolute numbers of stroke cases, disability, and death per year are increasing, with ischemic stroke accounting for about 85% (1, 2). Neurological deterioration after stroke is a severe clinical condition, resulting in poor long-term outcome and increased mortality. Therefore, early identification of neurological deterioration can assist in monitoring and individualised treatment of stroke when interventions are most effective. Neuroimaging is widely used in the diagnosis and assessment of stroke, and diffusion weighted imaging (DWI) of magnetic resonance imaging (MRI) enables the early and accurate diagnosis of ischemic stroke (2, 3). Diffusion tensor imaging (DTI) technique is more accurate at predicting the prognosis of motor injury when stroke directly damages the corticospinal tract (4). However, the pathologic mechanisms of ischemic stroke are complex, and it is currently recognised that the mechanisms of its occurrence and development include metabolic disorders, inflammation, penumbral depolarization, oxidative stress, calcium overload, and apoptosis (5). Thus, new peripheral blood biomarkers are also being actively explored for the long-term efficacy evaluation after the onset in order to provide clues for the mechanism of post-stroke reperfusion injury and explore new intervention targets for neuroprotective treatment.

At present, the diagnosis and assessment of stroke mainly rely on clinical evaluation and neuroimaging, so more convenient, rapid, and accurate haematological markers are helpful to improve or speed up the diagnosis process of stroke so as to facilitate the earlier intervention treatment (6). Non-coding RNAs (ncRNAs), the products of eukaryotic transcription, mainly consist of microRNAs (miRNAs), long non-coding RNAs (lncRNAs) and circular RNAs (circRNAs) (7). As a member of non-coding RNA family, circRNAs are generated by head-to-tail splicing and form a closed-loop structure, making them more stable and conserved (8, 9). Accumulated evidence indicated that circRNAs regulate gene expression through several approaches including miRNA sponges (10), RNA-binding proteins sponges (11), transcriptional activators (12), and translation into proteins (13). Compared with miRNA and lncRNA, circRNAs are more stable and not easy to degrade in peripheral blood, and their potential role in regulating synaptic function and neuroplasticity makes them a hot research spot in the field of stroke in recent years (14).

Our previous study found that the expression of three circRNAs [circular RNA FUNDC1 (circFUNDC1), circular RNA PDS5B (circPDS5B), and circular RNA CDC14A (circCDC14A)] was higher in acute ischemic stroke (AIS) patients than controls, and the levels of three circRNAs could predict stroke outcomes, which demonstrated that these three circRNAs could be served as potential biomarkers for the diagnosis and prognosis of AIS (15). Thus, the present study aimed to figure out the relationship between circRNAs and acute neurological deterioration and long-term survival, which could be helpful to guide the therapy and secondary prevention of acute ischemic stroke.

## Methods

### Ethics Statement

The study protocol was approved by the Research Ethics Committee of the Affiliated ZhongDa Hospital, Southeast University (Approval ID: 2016-SR-235). Written informed consents were obtained from all the participants or their legally authorised representatives.

### Patients

Patients diagnosed with AIS were recruited through the Neurology Department of ZhongDa Hospital from November 2017 to February 2019. Healthy controls were enrolled from the physical examination centres matched by age, gender, and education level during the same period. The diagnosis of AIS was defined by an acute neurological deficit confirmed with MRI or computed tomography scan.

All included patients had a duration of fewer than 72 h from the onset of stroke symptoms to hospital admission. Exclusion criteria of the patients were as follows: (1) active malignant diseases; (2) other neurological and psychiatric diseases; (3) patients undergoing surgery within the last 3 months; (4) patients who used unfractionated heparin or low molecular weight heparin within the last month. In addition, aetiology of ischemic stroke was classified according to the Trial of Org 10172 in Acute Stroke Treatment (TOAST) criteria (16). The severity of ischemic stroke was evaluated by the National Institutes of Health Stroke Scale (NIHSS), which was assessed by experienced neurologists during the hospitalisation. Early neurological deterioration (END) was defined as an increase in the total NIHSS score of 2 or more within 72 h of stroke onset (17–19).

### Collection of Participants' Characteristics

Clinical data of the patients and controls were collected, including gender, age, histories of hypertension, diabetes mellitus, hyperlipemia, smoking, and previous transient ischemic attack (TIA). Meanwhile, the data of blood routine, biochemical indexes, and fibrinolytic parameters were also recorded.

### Follow up of AIS Patients

The modified Rankin Scale (mRS) score at 14 and 90 days after onset were followed up by telephone or outpatient service. The outcome of ischemic stroke was assessed by the mRS score. For the convenience of analysis, mRS was divided into good outcome (mRS 0–2) and poor outcome (mRS 3–6). Patients were followed until death or the end of 18 months of follow-up, and the time of death and cause of death were recorded. The long-term outcome was survival, defined as the time from the date of stroke diagnosis to death, or until the end of an 18-month follow-up for surviving patients.

### RNA Extraction and qPCR Assay

According to our previous study, three circRNAs were proved to be elevated in AIS patients through a 3-stage approach involving discovery, validation, and replication (15). Briefly, peripheral blood samples from AIS patients were collected at the arrival of hospital, and the whole blood was drawn into EDTA tubes (BD Biosciences) prior to any therapy. Then, the blood samples were centrifuged at 1,000 g for 10 min at 4°C. The total plasma RNA was extracted from the blood samples using the miRNeasy Mini kit (Qiagen) as directed by the manufacturer. The concentration of total RNA was evaluated by a NanoDrop ND-1000 spectrophotometer (Thermo Fisher Scientific, Waltham, MA, USA). Then, RNA was reverse transcribed to cDNA by the HiScript Q RT SuperMix for qPCR Kit (Vazyme, R123-01) with the manufacturer's instructions. Subsequently, quantitative PCR was implemented by using SYBR Green Real-time PCR Master Mix (Vazyme, R131-01) on the Applied Biosystems QuantStudio 6 (Applied Biosystems) following the manufacturer's recommended cycling conditions. All samples were performed in duplicate. Finally, circRNA copy values were calculated according to the standard curve, Ct value, and RNA concentration. Primers applied to qPCR were as follows: circFUNDC1, forward: CCATCTGAAGCTTGGCAAACT; reverse: TTCAACTCTCTTCCAGTCAATCT; circPDS5B, forward: ATTGCTCTCCTTGCACCTGA, reverse: TCGCATGGATACAATGAATGGC; and circCDC14A, forward: CCATTCTCGACTGTTTGCAGG, reverse: GACAGGAGTGCTCTGTAGGC.

### Statistical Analysis

Statistical analysis was performed using Statistical Packages for Social Sciences (SPSS) statistical software version 25.0 (IBM). Continuous data were displayed as mean ± standard deviation (SD) or median (interquartile range, IQR) and categorical data were expressed as number (percentage). The Kolmogorov-Smirnov test was used to assess the normality. The normally distributed data of the two groups used independent sample *t*-test. In the skewed distributed data, the Mann-Whitney *U-*test was applied to analyse the difference between two groups, and the Kruskal-Wallis *H-*test was used to test the difference between multiple groups. Categorical variables were analysed by Chi-square test or Fisher's exact test. Bonferroni correction was used to correct the significance level for multiple comparisons. Correlation analysis was performed using Spearman's rank correlation test. The interactive effect was assessed by multivariate linear regression. Logistic regression analysis was used to test the relationship between circFUNDC1 and END. Receiver operating characteristic (ROC) curve analysis was performed by assessing the ability of the circFUNDC1 to predict END. Data were subjected to survival analyses according to Kaplan-Meier curves and log-rank tests. Statistical significance was set as *p* < 0.05.

## Results

### The Baseline Characteristics of the Subjects

**Figure 1:**
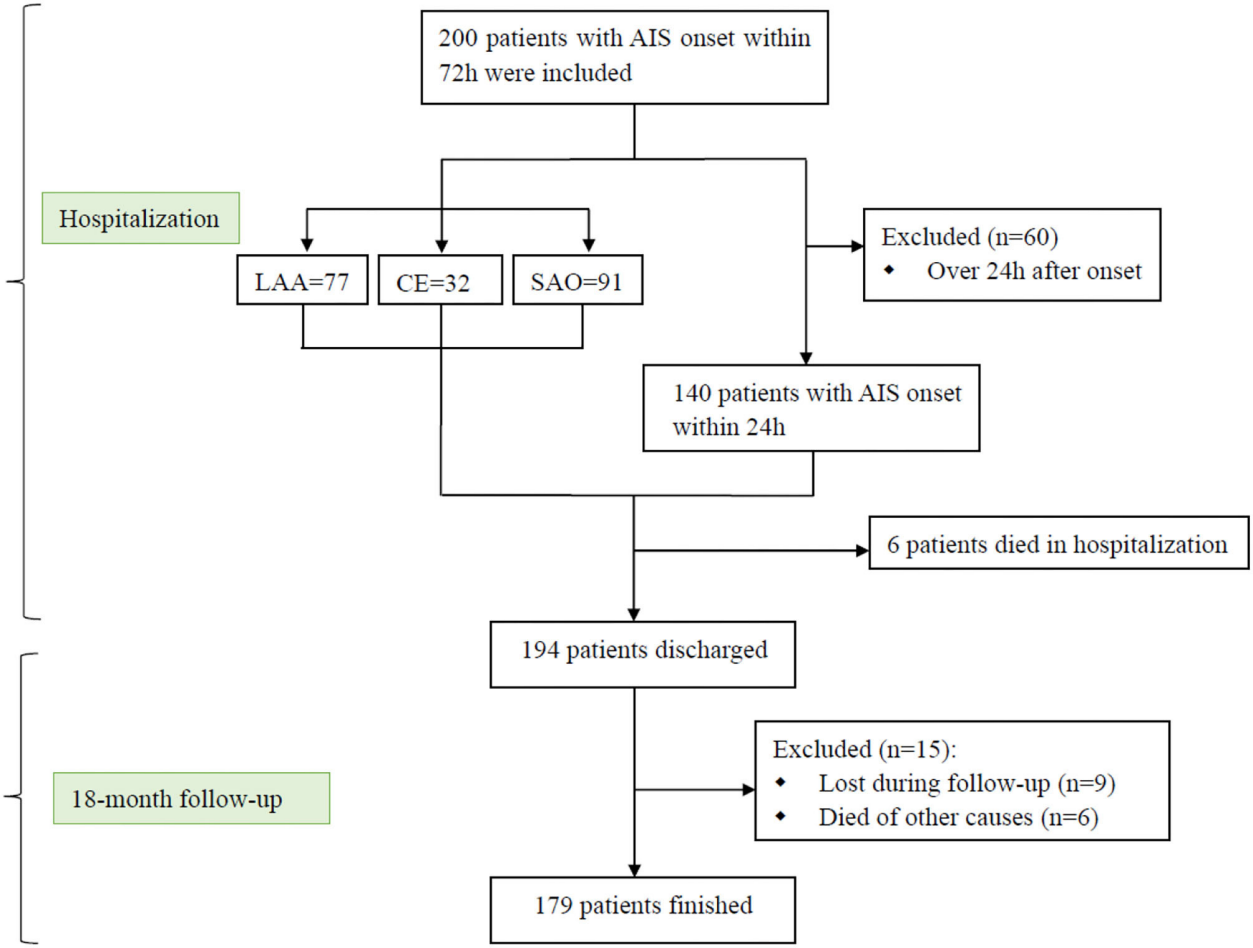
Research flow chart. AIS, acute ischemic stroke; LAA, large artery atherosclerosis; CE, cardioembolism; SAO, small artery occlusion.

A total of 200 patients and 100 healthy controls were included in the study. The stroke patients were further divided into three groups according to TOAST: large artery atherosclerosis (LAA, *n* = 77), cardioembolism (CE, *n* = 32), and small artery occlusion (SAO, *n* = 91). The baseline demographic and clinical characteristics of the AIS patients with different stroke subtypes and controls are displayed in Supplementary Table S1. In addition, of all the patients included, 140 of them were admitted to the hospital within 24 h of onset, and 13 of them had early neurological deterioration. Demographics and baseline characteristics of the 140 patients are summarised in Table 1. The baseline NIHSS scores and mRS scores showed no significant difference between patients with END and those without END. However, the outcomes of patients without END were better than those who had END (*P* < 0.05). As shown in Figure 1, 6 patients died during the hospitalisation and 9 patients were lost to follow-up. Besides, 32 patients (16%) died of fatal stroke, and 6 patients died of other causes, such as pneumonia (2), sepsis (2), myocardial infarction (1), and hepatic failure (1).

**Table 1:** The general characteristics of stroke patients with END and without.

	**Non-END (*n* = 127)**	**END (*n* = 13)**	***P***
**Demographic characteristics**			
Male, *n* (%)	86 (67.7)	12 (92.3)	0.127
Age, median (IQR), years	72 (21)	78 (15)	0.628
**Vascular risk factors**			
Hypertension, *n* (%)	95 (74.8)	10 (76.9)	1.000
Diabetes mellitus, *n* (%)	40 (31.5)	3 (23.1)	0.756
Hyperlipemia, *n* (%)	30 (23.6)	5 (38.5)	0.401
Smoking, *n* (%)	31 (24.4)	5 (38.5)	1.441
Previous TIA, *n* (%)	23 (18.1)	2 (15.4)	1.000
**Laboratory parameters**			
SBP, median (IQR) (mmHg)	153.5 (33)	170 (15)	0.239
DBP, mean ± SD (mmHg)	85.65 ± 16.09	89.92 ± 15.11	0.360
TC, mean ± SD (mmol/L)	4.53 ± 1.43	4.56 ± 1.43	0.934
TG, median (IQR) (mmol/L)	1.10 (0.72)	1.29 (1.05)	0.780
LDL, mean ± SD (mmol/L)	2.69 ± 0.83	2.83 ± 1.12	0.591
HDL, median (IQR) (mmol/L)	1.13 (0.33)	1.00 (0.35)	0.394
Creatinine, median (IQR) (μmol/L)	77 (33)	68 (26)	0.042
BUN, median (IQR) (mmol/L)	5.65 (2.4)	5.1 (1.8)	0.194
TP, median (IQR) (g/L)	63.8 (8.3)	66.6 (13.2)	0.720
Albumin, median (IQR) (g/L)	38.4 (4.6)	40.0 (6.9)	0.688
WBC, median (IQR) (10^9^/L)	7.02 (3.06)	8.45 (4.88)	0.233
RBC, median (IQR) (10^12^/L)	4.59 (0.80)	4.76 (0.76)	0.827
PLT, mean ± SD (10^9^/L)	194.43 ± 63.25	220.54 ± 46.07	0.151
Haemoglobin, median (IQR) (g/L)	139 (25)	143 (21)	0.738
Lipoprotein -a, median (IQR) (mg/L)	224 (358)	216 (283)	0.769
**TOAST**			0.017
LAA, *n* (%)	50 (39.4)	10 (76.9)	
CE, *n* (%)	25 (19.7)	2 (15.4)	
SAO, *n* (%)	52 (40.9)	1 (7.7)	
**Medication**			
ACE I/ARBs, *n* (%)	28 (22.0)	1 (7.7)	0.391
β-Blockers, *n* (%)	7 (5.5)	1 (7.7)	1.000
Calcium channel blockers, *n* (%)	40 (31.5)	1 (7.7)	0.140
Diuretics, *n* (%)	16 (12.6)	0 (0)	0.367
**Follow up**			
NIHSS (baseline), median (IQR)	4 (6)	9.5 (12)	0.127
NIHSS (7th day), median (IQR)	2 (5)	12.5 (9)	<0.001
mRS (baseline), median (IQR)	3 (3)	4 (4)	0.282
mRS (7th day), median (IQR)	1.5 (3)	5 (1)	<0.001
mRS (14th day), median (IQR)	1 (3)	5 (1)	<0.001
mRS (90th day), median (IQR)	1 (3)	4.5 (1)	<0.001

*END, early neurological deterioration; SD, standard deviation; IQR, interquartile range; SBP, systolic blood pressure; DBP, diastolic blood pressure; TC, total cholesterol; TG, triglyceride; LDL, low density lipoprotein cholesterol; HDL, high density lipoprotein cholesterol; BUN, blood urea nitrogen; TP, total protein; WBC, white blood cells; RBC, red blood cells; PLT, platelets; ACE I, angiotensin-converting enzyme inhibitor; ARBs, angiotensin II receptor blockers; LAA, large artery atherosclerosis; CE, cardioembolism; SAO, small artery occlusion; NIHSS, National Institutes of Health Stroke Scale; mRS, modified Rankin Scale*.

### Levels of circRNAs in Different Stroke Subtypes and Controls

**Figure 2:**
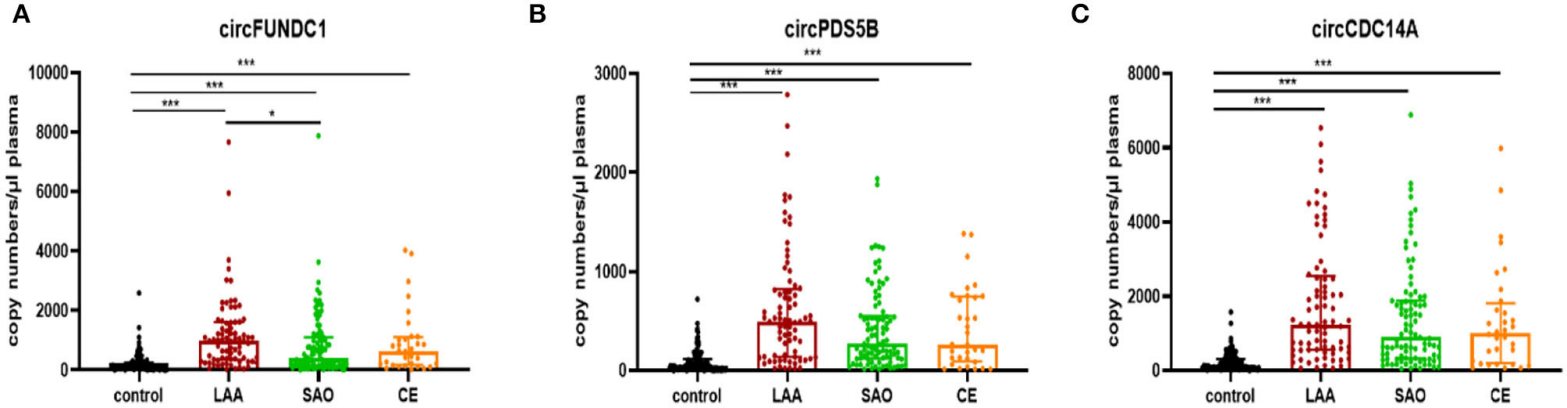
Levels of circRNAs in acute ischemic stroke patients (AIS) with different TOAST subtypes. The plasma concentrations of circFUNDC1 **(A)**, circPDS5B **(B)**, and circCDC14A **(C)** from AIS patients were profiled in the TOAST categories large artery atherosclerosis (LAA), small artery occlusion (SAO), and cardioembolism (CE). TOAST, Trial of Org 10172 in Acute Stroke Treatment. Median ± interquartile range, Kruskal-Wallis H-Test. ^***^P < 0.001; ^*^P < 0.05.

Levels of all three circRNAs (circFUNDC1, circPDS5B, and circCDC14A) were significantly increased in LAA stroke, SAO stroke, and CE stroke patients compared to the controls (*P* < 0.001; Figure 2). However, there was no significant difference in further subgroup analysis, with an exception that the levels of circFUNDC1 in LAA stroke patients were significantly higher than SAO stroke patients [LAA vs. SAO, 984.37 (1,264.74) vs. 413.93 (956.36), *P* = 0.015; Figure 2A].

### The Relationship Between NIHSS Scores, mRS Scores, and Levels of circRNAs in Different Stroke Subtypes

**Figure 3:**
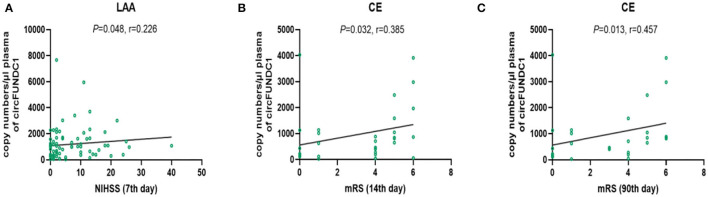
The relationship between circFUNDC1 levels and NIHSS scores and mRS scores in acute ischemic stroke (AIS) patients. The scatterplot showed circFUNDC1 levels were positively associated with NIHSS scores on the 7th day in LAA stroke patients **(A)**, mRS scores on the 14th day **(B)**, and the 90th day **(C)** in CE stroke patients. NIHSS, National Institutes of Health Stroke Scale; mRS, modified Rankin Scale; LAA, large artery atherosclerosis; CE, cardioembolism.

Although the levels of these three circRNAs were not associated with NIHSS scores and mRS scores in the overall patients, we found that circFUNDC1 levels were associated with these scores in specific subtypes of patients. The circFUNDC1 levels positively correlated with NIHSS scores on the 7th day in LAA stroke patients (*P* = 0.048, *r* = 0.226; Figure 3A), mRS scores both on the 14th day (*P* = 0.032, *r* = 0.385; Figure 3B), and the 90th day (*P* = 0.013, *r* = 0.457; Figure 3C) in CE stroke patients. However, the levels of these circRNAs were not correlated with NIHSS scores or mRS scores in SAO stroke patients.

### Kaplan–Meier Curves for Survival in AIS Patients

**Figure 4:**
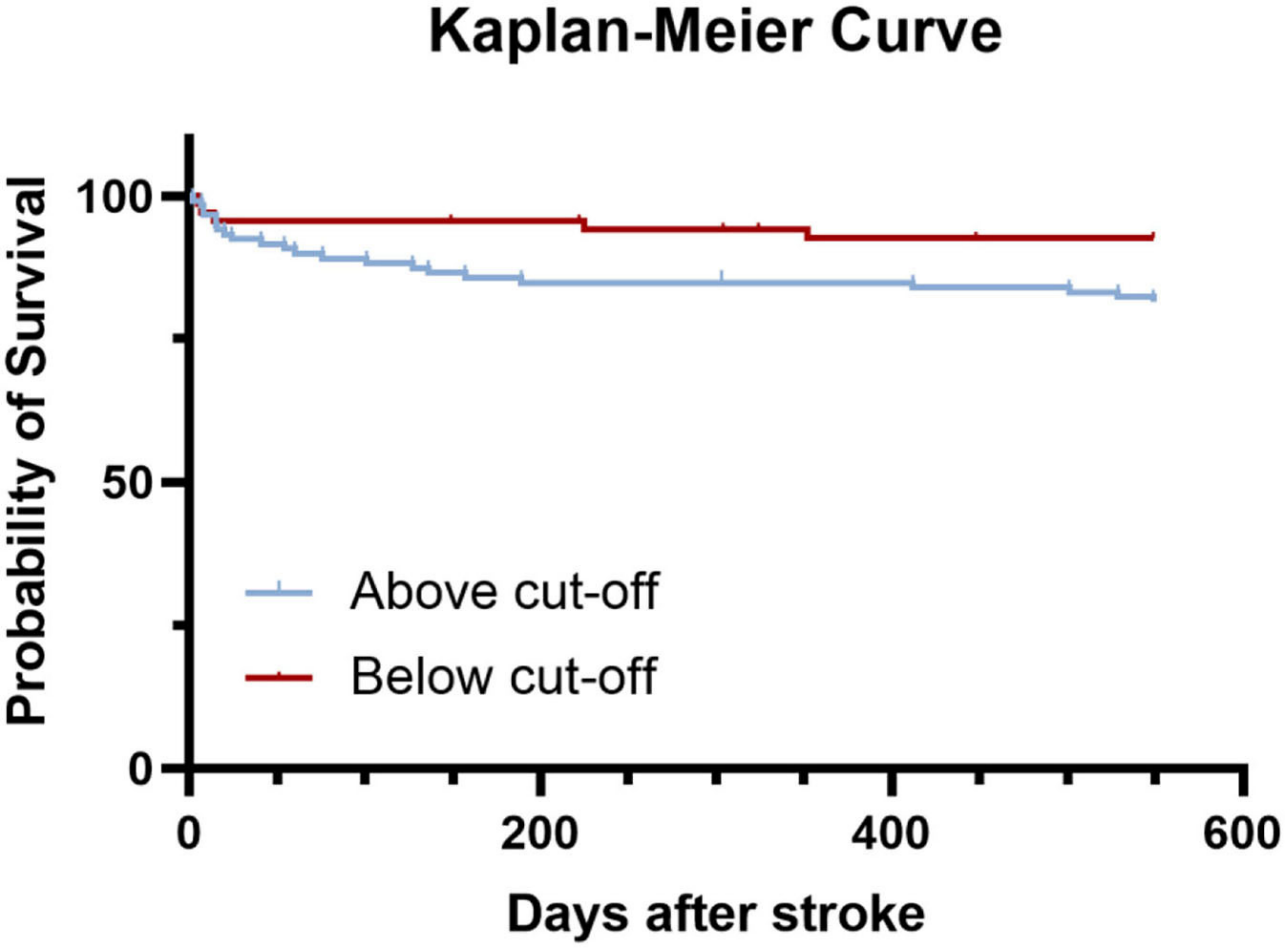
Kaplan–Meier curves estimated survival up to 18 months (549 days) after stroke. Log rank test, *P* = 0.042.

To further verify the significance of circFUNDC1 in stroke long-term outcome, we followed the patients for 18 months after stroke onset. Our previous study showed that circRNAs could predict stroke outcome at 3 months, thus cut off value of the ROC curve between circFUNDC1 levels and mRS score at 3 months was defined as “354.30 copies/μl plasma”, with sensitivity at 81.4% and specificity at 45.4%, respectively (15). Furthermore, the survival Kaplan-Meier curves of AIS patients with low (below cut-off) or high circFUNDC1 levels (above cut-off) were constructed after 18 months of follow-up, and there was a significant difference between the two groups (Figure 4, *P* = 0.042). However, levels of circPDS5B and circCDC14A showed no significance in survival for AIS patients.

### Levels of circRNAs in Patients With and Without Early Neurological Deterioration Who Were Admitted to Hospital Within 24 h of Onset

**Figure 5:**
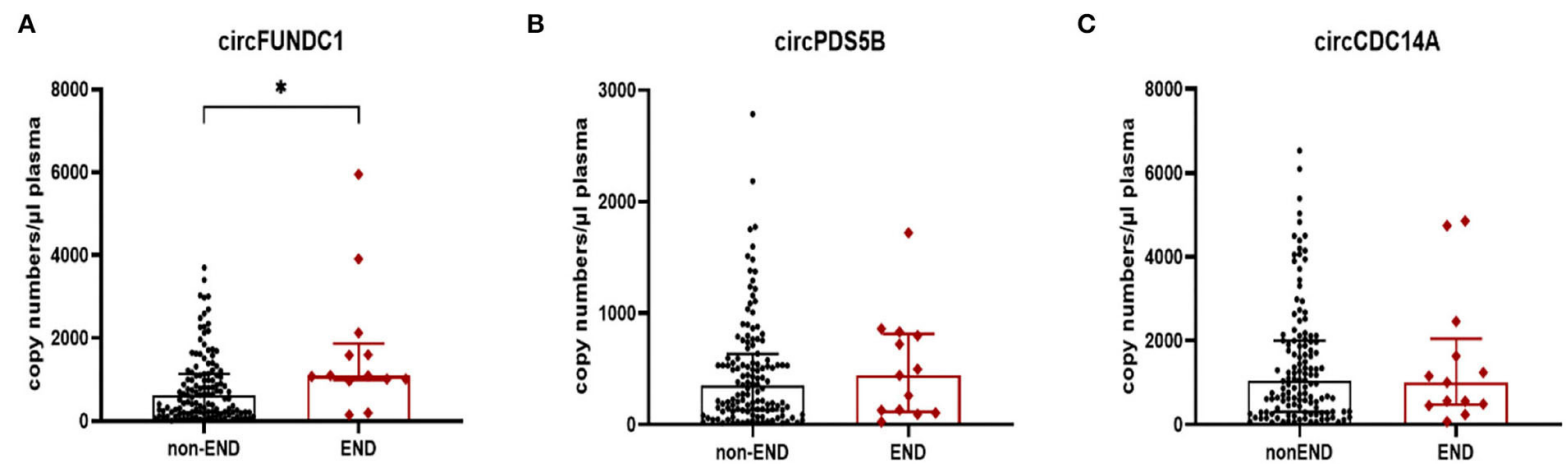
Expression of circFUNDC1 **(A)**, cricPDS5B **(B)**, and circCDC14A **(C)** in acute ischemic stroke (AIS) patients. END, early neurological deterioration. Median ± interquartile range, Mann-Whitney test. ^*^P < 0.05.

The levels of circFUNDC1 in patients with END were significantly higher than those without END (Figure 5A, *P* = 0.013). However, no significant difference was observed for circPDS5B (Figure 5B, *P* = 0.722) and circCDC14A (Figure 5C, *P* = 0.951).

### ROC Curve for the Prediction of END in AIS Patients Admitted to Hospital Within 24 h of Onset

**Figure 6:**
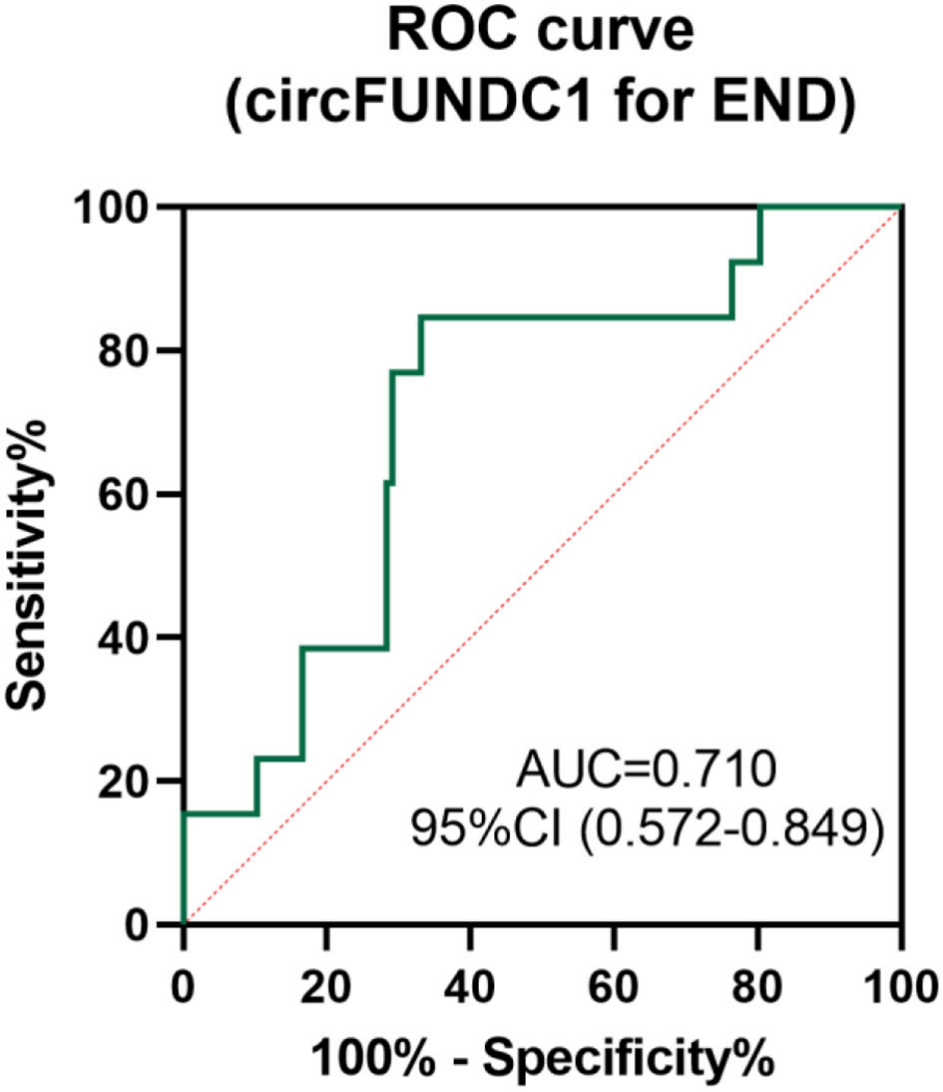
Receiver operating characteristic (ROC) curve for the value of circFUNDC1 to predict early neurological deterioration (END). AUC, area under the curve; CI, confidence interval.

Logistic regression revealed circFUNDC1 levels remained associated with early neurological deterioration when adjusted for age, vascular risk factors, baseline NIHSS, and stroke associated infection (SAI) (Table 2, odds ratio: 1.001, *P* = 0.005). To investigate the clinical value of circFUNDC1 in predicting END, a ROC curve was performed to compare the levels of circFUNDC1 between END and non-END patients (Figure 6), which yielded a sensitivity of 84.6% and a specificity of 66.9%, with the area under the curve (AUC) at 0.710 (95% CI, 0.572–0.849).

**Table 2:** Association between circFUNDC1 and early neurological deterioration.

	**OR**	**95% CI**	***P***
Age	1.027	0.970–1.087	0.357
Hypertension	1.413	0.283–7.052	0.673
Diabetes mellitus	0.843	0.175–4.053	0.831
Hyperlipemia	2.319	0.535–10.049	0.261
Smoking	4.671	0.850–25.659	0.076
Previous TIA	1.318	0.205–8.469	0.771
NIHSS (baseline)	0.977	0.914–1.092	0.999
SAI	5.968	1.093–32.575	0.039
circFUNDC1	1.001	1.000–1.001	0.005

*NIHSS, National Institutes of Health Stroke Scale; SAI, stroke associated infection; OR, odds ratio; CI, confidence interval*.

### The Relationship Between NIHSS Scores, mRS Scores, and Levels of circRNAs in AIS Patients Admitted to Hospital Within 24 h of Onset

**Figure 7:**
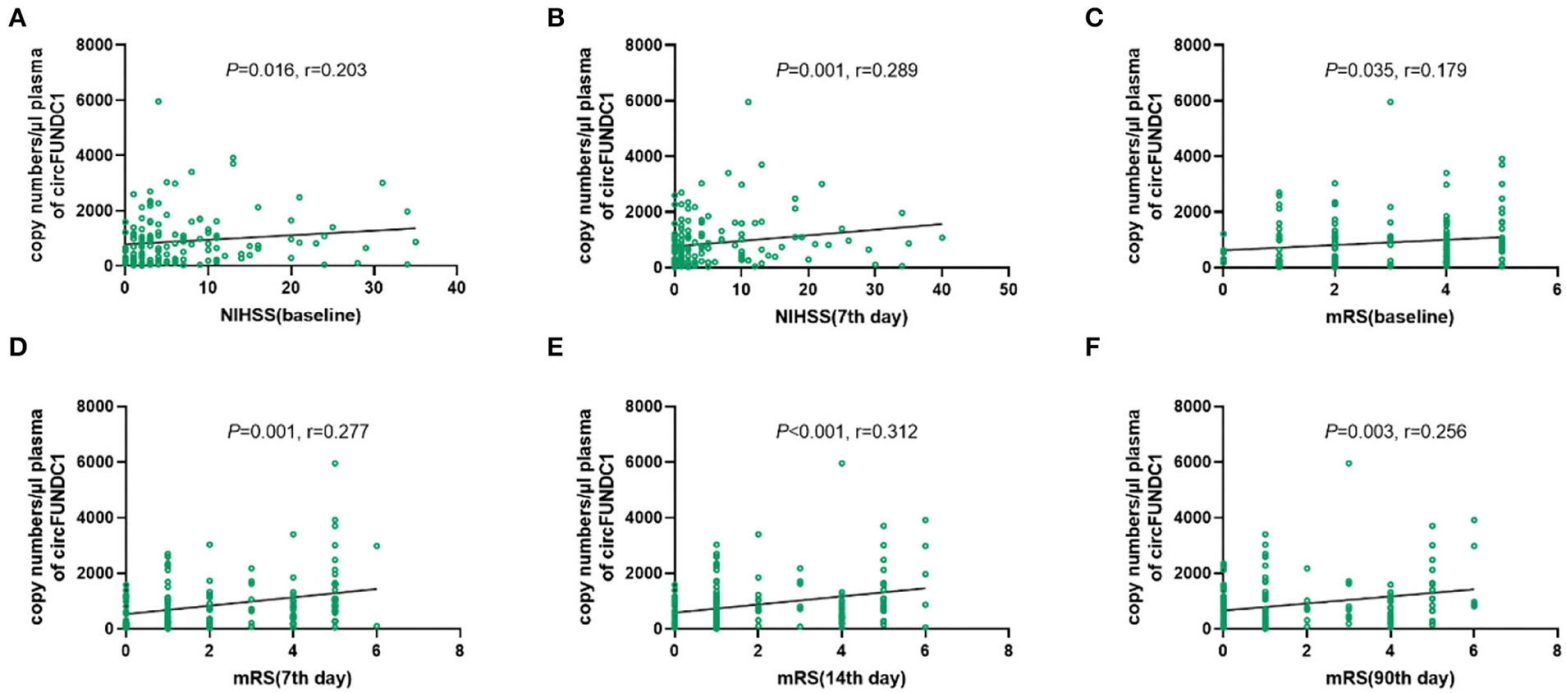
The relationship between NIHSS scores, mRS scores, and expression of circFUNDC1 in acute ischemic stroke (AIS) patients within 24h after onset. The scatterplot showed circFUNDC1 levels were positively associated with baseline NIHSS scores **(A)**, the 7th day NIHSS scores **(B)**, baseline mRS scores **(C)**, the 7th day mRS scores **(D)**, the 14th day mRS scores **(E)**, and the 90th day mRS scores **(F)**. NIHSS, National Institutes of Health Stroke Scale; mRS, modified Rankin Scale.

There was a positive correlation between circFUNDC1 levels and baseline NIHSS scores (Figure 7A, *P* = 0.016, *r* = 0.203) and the 7th day NIHSS scores (Figure 7B, *P* = 0.001, *r* = 0.289) in patients admitted within 24 h after onset. Besides, a positive correlation was observed between circFUNDC1 levels and followed mRS scores (Figure 7, baseline: *P* = 0.035, *r* = 0.179; the 7th day: *P* = 0.001, *r* = 0.277; the 14th day: *P* < 0.001, *r* = 0.312; the 90th day: *P* = 0.003, *r* = 0.256). However, the levels of circPDS5B and circCDC14A were not associated with NIHSS scores and mRS scores in patients admitted within 24 h after onset.

### Interaction of circFUNDC1 and NLR in AIS Patients Admitted to Hospital Within 24 h of Onset

**Figure 8:**
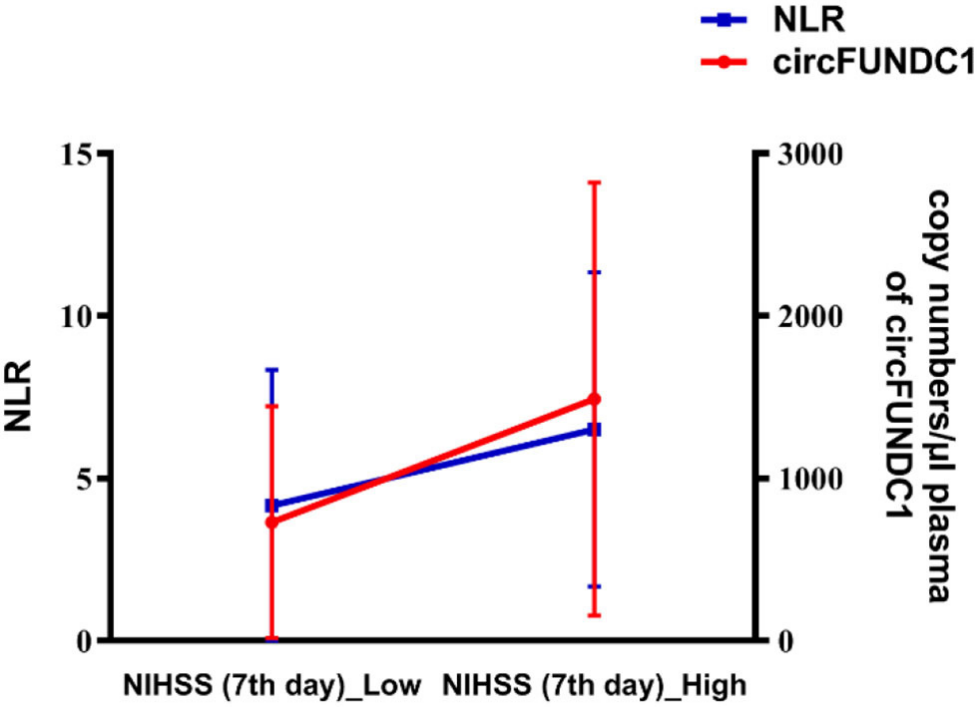
Interactive effect of circFUNDC1 and NLR (neutrophil to lymphocyte ratio) on the seventh NIHSS scores in patients admitted within 24 h after onset. NIHSS, National Institutes of Health Stroke Scale. Multiple linear regression analysis, *P* < 0.001.

Multiple linear regression analysis verified the correlations between circFUNDC1 levels and the 7th day NIHSS scores in patients admitted to hospital within 24 h of onset (*P* = 0.026, *R*^2^ = 0.036). Further interaction analysis showed that both higher levels of circFUNDC1 and neutrophil to lymphocyte ratio (NLR) represented more severe neurological function symptoms (Figure 8, standardised $\beta_{\text{circFUNDC}1^{*}\text{NLR}}$ = 0.432, *P* < 0.001) in patients admitted within 24 h after onset.

## Discussion

In the present study, we demonstrated that: (1) Plasma levels of three circRNAs increased in patients with different ischemic stroke subtypes compared to healthy controls. The circFUNDC1 levels of LAA stroke patients were significantly higher than those of SAO stroke patients; (2) the levels of circFUNDC1 were significantly higher in patients with END than those without; (3) the levels of circFUNDC1 within 24 h of onset were positively correlated with the patient's baseline and the 7th day NIHSS score, however, the levels of circFUNDC1 within 72 h of onset were only positively correlated with the 7th day NIHSS score in LAA patients; (4) the patients with high levels of circFUNDC1 had a shorter post-stroke survival at 18 months after stroke.

In this study, plasma circRNAs levels were analysed in different ischemic stroke subtypes according to TOAST criterion. Besides, considering that the levels of circRNAs change in peripheral blood overtime after the onset of stroke, we selected circRNAs levels of patients admitted within 24 h of the onset for further analysis. Moreover, to the best of our knowledge, this is the first study to analyse the relationship between circRNAs and early neurological deterioration in AIS patients.

A number of studies have reported the change levels of circRNAs in stroke patients, but few studies have explored the relationship between circRNAs and etiological subtypes of ischemic stroke. Furthermore, only a previous study reported different stroke subtypes expressing specific circRNAs profiles, and they found the levels of has_circRNA_102488 were higher in cardioembolism stroke compared to atherothrombotic stroke (20). Our present study also showed circFUNDC1 elevated differently according to the etiological subtypes in AIS patients.

Besides, we found that END was associated with the etiological subtypes of stroke, and the prognosis of the patients with END was worse than that of the non-END, which were consistent with previous studies (21, 22). Mounting researches are exploring reliable biomarkers for predicting neurological deterioration after AIS, yet none has been recommended for clinical use (23). However, to our knowledge, this study is the first attempt to figure out whether circRNAs could be the potential predictor for END. Thus, we found circFUNDC1 levels in patients with END were significantly higher than those without END, and a ROC curve indicated the predictive value of circFUNDC1 for END.

In the present study, we also found the levels of circFUNDC1 within 24 h of onset were positively correlated with NIHSS score in AIS patients. However, the levels of circFUNDC1 within 72 h of onset were only positively correlated with the 7th day NIHSS score in LAA patients. This suggested that circFUNDC1 was more effective in predicting the severity of neurological deficits in LAA patients. Moreover, the changing trend of circFUNDC1 in stroke patients with good outcome was opposite to that of poor outcome patients (15). Therefore, the immediate detection of peripheral blood circFUNDC1 levels within 24 h of the onset of AIS patients can better reflect the severity and outcome of neurological deficits. However, the positive correlations between NIHSS scores, mRS score, and levels of circFUNDC1 appear to be weak due to the low r-value. Nevertheless, it may suggest a clue in which patients with high circFUNDC1 levels might develop more severe neurological deficits during hospitalisation. Therefore, large independent samples of patients should be repeatedly verified in further research.

Besides, our study found a positive correlation between NLR and NIHSS scores, which was consistent with previous studies (24–27). Additionally, we found an interactive effect of circFUNDC1 and NLR levels on NIHSS scores, suggesting both higher circFUNDC1 and NLR levels indicated more severe neurological deficits.

As reported in our previous study, neutrophils might be important origins of elevated circFUNDC1 levels in the plasma of AIS patients (28). In addition, FUNDC1, encoded by the host gene of circFUNDC1, has been verified to contribute to hypoxia-induced mitophagy as a mitochondrial membrane protein (29). Furthermore, autophagy activation aggravates ischemic brain injury in certain circumstances (30). These may partially explain the more severe symptoms of neurological deficits in patients with higher levels of circFUNDC1, but further experiments are needed to explore the precise mechanisms in the future.

In our work, the in-hospital death rate was 3%, similar to the study from bigdata observatory platform for stroke of China, ranging from 0.9 to 5.1% (31). However, the previous research recruited first-ever stroke patients, which may cause the difference in results. Furthermore, another interesting finding of the present study was that levels of circFUNDC1 could predict long-term survival after stroke. Thus, these results showed that circFUNDC1 had predictive value in both the acute phase and the long-term prognosis of acute ischemic stroke.

There are also other multiple biomarkers in predicting stroke outcomes. For instance, the levels of C reactive protein (CRP) were higher in poor outcome patients (32), which was consistent with our results (Supplementary Figure S1). However, there was no correlation between levels of CRP and circFUNDC1 (Supplementary Figure S2). Although various biomarkers have been suggested to be associated with the prognosis of ischemic stroke, none of them has been directly used clinically due to the impact of other clinical variables and the limitations of measurement methodologies (33, 34). Nevertheless, these biomarkers do have unique advantages and attractiveness, such as providing pathological mechanisms leading to poor outcomes and exploring new therapeutic strategies (35, 36).

To sum up, the present study was novel in predicting END during hospitalisation and survival time after discharge using circFUNDC1 levels at admission. Besides, the current work performed a subgroup analysis of circFUNDC1 levels in stroke patients with different TOAST classifications. Improving the prognosis of stroke has always been the priority in the course of the disease. China Stroke Prevention Project Committee (CSPPC) stroke program turns out to be an efficient strategy in enhancing stroke care to improve the prognosis (37). Moreover, our present study found that patients with higher circFUNDC1 levels at admission had more severe neurological symptoms and shorter survival times, which indicated that these patients may need more comprehensive care in hospitalisation. Therefore, peripheral blood circFUNDC1 levels detection can be used as a time-effective pre-screening tool for identifying individuals with high risk for END and fewer survival times. In a word, early and accurate identification of stroke prognosis is beneficial to stroke management, such as anticipated quality of life and survival time, which might influence treatment decisions.

There were some limitations in our study. First, selection bias in this study was inevitable. Second, as a small sample study, there were too few patients with stroke of other determined aetiology (ODE) and undetermined aetiology (UDE) to be included in the study. Third, although the risk of poor prognosis increased with the increase of circFUNDC1 levels after adjustment of treatment (Supplementary Table S2), more patients with different therapies should be involved to further verify the predictive role of circFUNDC1 in stroke outcomes, especially for subtype analysis of reperfusion therapies such as intravenous alteplase and endovascular therapy. Besides, the exact mechanism of stroke prognosis and circFUNDC1 expression was unknown, which needs further *in vitro* and *in vivo* experiments to investigate the detailed mechanism.

## Conclusion

We demonstrated that elevated circFUNDC1 levels differed among the etiological types of stroke, and circFUNDC1 levels were higher in patients with END. We also demonstrated that immediate detection of circFUNDC1 levels in peripheral blood within 24 h of the onset of stroke could better reflect the severity and prognosis of neurological dysfunction. Patients with higher circFUNDC1 levels had a shorter survival time. Therefore, circFUNDC1 could be a potential predictor of acute-phase outcome and long-term survival of acute ischemic stroke.

## Data Availability Statement

The raw data supporting the conclusions of this article will be made available by the authors, without undue reservation.

## Ethics Statement

The studies involving human participants were reviewed and approved by Research Ethics Committee of the Affiliated ZhongDa Hospital, Southeast University (Approval ID: 2016-SR-235). The patients/participants provided their written informed consent to participate in this study.

## Author Contributions

ZZ, JZ, and LZu designed the study and performed the statistical analysis. JZ, LZu, LZh, and ZW collected patient information and conducted experiment. JZ, YS, and LG prepared the figures. JZ prepared the manuscript draft. All authors have read and approved the final manuscript.

## Funding

This work was supported by Jiangsu Provincial Medical Outstanding Talent (JCRCA2016006).

## Conflict of Interest

The authors declare that the research was conducted in the absence of any commercial or financial relationships that could be construed as a potential conflict of interest.

## Publisher's Note

All claims expressed in this article are solely those of the authors and do not necessarily represent those of their affiliated organizations, or those of the publisher, the editors and the reviewers. Any product that may be evaluated in this article, or claim that may be made by its manufacturer, is not guaranteed or endorsed by the publisher.

## Supplementary Material

Supplementary Figure 1 Levels of CRP **(A)** and circFUNDC1 **(B)** in patients with good and poor outcomes. CRP, C reactive protein. ^**^*P* < 0.01; ^*^*P* < 0.05.

Supplementary Figure 2 The correlation between circFUNDC1 and CRP. CRP, C reactive protein.
