# Supplementary material for: Circular RNA FUNDC1 for Prediction of Acute Phase Outcome and Long-Term Survival of Acute Ischemic Stroke
Source: Front Neurol. 2022 Jun 3;13:846198. doi: 10.3389/fneur.2022.846198 (PMC9203888; doi:10.3389/fneur.2022.846198)
Supplement: Supplementary Figure 1 — Levels of CRP (A) and circFUNDC1 (B) in patients with good and poor outcomes. CRP, C reactive protein. **P < 0.01; *P < 0.05. [file Data_Sheet_1.PDF]

Table S1 The general characteristics of patients with different ischemic stroke subtypes

Variables	Control (n=100)	LAA (n=77)	CE (n=32)	SAO (n=91)	*P*
**Demographic characteristics**					
Male, n (%)	53 (53)	54 (70.1)	18 (56.3)	63 (69.2)	0.071
Age, mean ± SD, years	61.4 ± 16.0	68.6 ± 13.4	75.9 ± 12.7	67.9 ± 12.9	<0.001
**Vascular risk factors**					
Hypertension, n (%)	45 (45)	58 (75.3)	24 (75)	70 (76.9)	<0.001
Diabetes mellitus, n (%)	10 (10)	22 (28.6)	8 (25)	35 (38.5)	<0.001
Hyperlipemia, n (%)	28 (28)	19 (24.7)	6(18.8)	29 (31.9)	<0.001
Smoking, n (%)	7 (7)	22 (28.6)	5 (15.6)	24 (26.4)	0.001
Previous TIA, n (%)	7 (7)	13 (16.9)	5 (15.6)	14 (15.4)	0.143
**Laboratory parameters**					
SBP, mean ± SD (mmHg)	139.7 ± 18.6	156.6 ± 22.8	156.4 ± 24.1	159.1 ± 27.9	<0.001
DBP, mean ± SD (mmHg)	79.7 ± 11.4	83.5 ± 14.0	85.6 ± 15.1	86.9 ± 16.4	0.019
TC, mean ± SD (mmol/L)	4.87 ± 0.92	4.63 ± 1.05	3.95 ± 0.97	4.69 ± 1.07	0.001
TG, median (IQR) (mmol/L)	1.36 (0.90)	1.14 (0.77)	1.00 (0.52)	1.28 (1.00)	0.033
LDL, mean ± SD (mmol/L)	2.91 ± 0.76	2.80 ± 0.86	2.24 ± 0.81	2.77 ± 0.77	0.002

HDL, median (IQR) (mmol/L)	1.22 (0.34)	1.14 (0.28)	1.03 (0.37)	1.14(0.30)	0.146
Creatinine, median (IQR) (μmol/L)	65.0 (20.0)	72.5 (26.0)	86.5 (32.0)	75.0 (29.0)	<0.001
BUN, median (IQR) (mmol/L)	5.4 (2.0)	5.0 (2.3)	5.9 (2.6)	5.9 (2.4)	0.071
TP, mean ± SD (g/L)	66.2 ± 5.1	64.7 ± 7.0	65.4 ± 4.6	65.5 ± 6.9	0.215
Albumin, median (IQR) (g/L)	41.0 (4.2)	38.4 (4.7)	37.6 (4.5)	39.5 (4.9)	<0.001
WBC, median (IQR) (10^9/L)	6.3 (2.6)	7.4 (3.3)	7.2 (4.4)	6.5 (2.5)	0.002
RBC, median (IQR) (10^{12}/L)	4.5 (0.6)	4.6 (0.9)	4.7 (1.1)	4.7 (0.8)	0.007
PLT, median (IQR) (10^9/L)	202 (81)	215 (89)	178 (93)	201 (74)	0.399
Hemoglobin, median (IQR) (g/L)	134 (20)	140 (29)	141 (33)	140 (23)	0.023
Lipoprotein -a, median (IQR) (mg/L)	142 (362)	220.5 (440)	195.5 (249)	210.0 (302)	0.315
**Medication**					
ACE I/ARBs, n (%)	17 (17)	19 (24.7)	7 (21.9)	22 (24.2)	0.602
β-Blockers, n (%)	3 (3)	3 (3.9)	5 (15.6)	9 (9.9)	0.034
Calcium channel blockers, n (%)	23 (23)	27 (35.1)	6 (18.8)	29 (31.9)	0.172
Diuretics, n (%)	6 (6)	8 (10.4)	4 (12.5)	11 (12.1)	0.437

LAA, large artery atherosclerosis; CE, cardioembolism; SAO, small artery occlusion; SD, standard deviation; IQR, interquartile range; SBP, systolic blood pressure; DBP, diastolic blood pressure; TC, total cholesterol; TG, triglyceride; LDL, low density lipoprotein cholesterol; HDL,

high density lipoprotein cholesterol; BUN, blood urea nitrogen; TP, total protein; WBC, white blood cells; RBC, red blood cells; PLT, platelets; ACE I, angiotensin-converting enzyme inhibitor; ARBs, angiotensin II receptor blockers

Table S2 Logistic regression analysis for AIS outcome at 3 months

	OR	95%CI	*P*-value
circFUNDC1	1.001	1.000-1.001	0.010
Treatment			
non-reperfusion therapy	1		
reperfusion therapies	2.023	0.796-5.144	0.139

AIS, acute ischemic stroke; OR, odds ratio; CI, confidence interval.

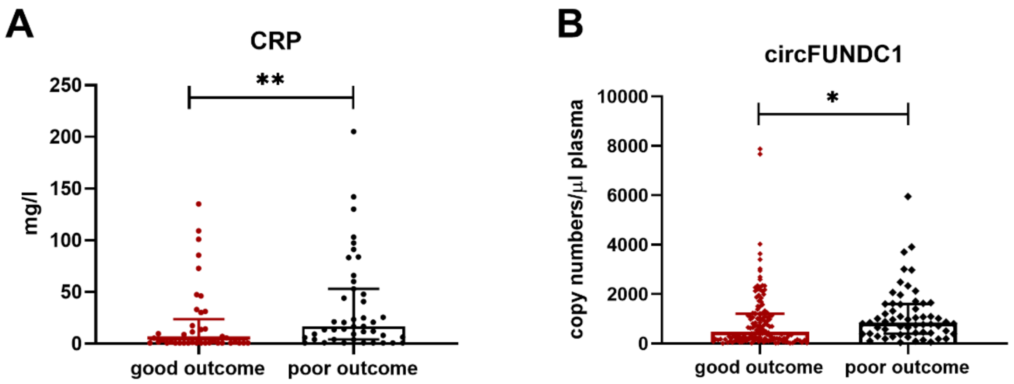

Figure S1 Levels of CRP and circFUNDC1 in patients with good and poor outcomes.

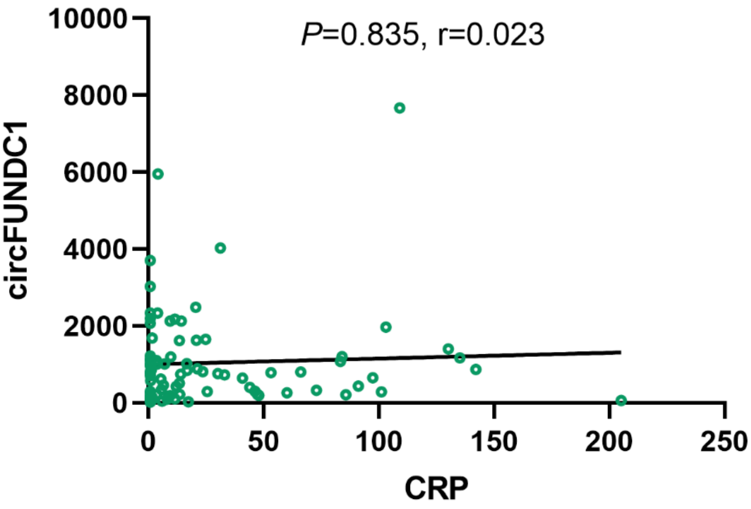

Figure S2 The correlation between circFUNDC1 and CRP.
